# Diagnostic and prognostic values of AKR1C3 and AKR1D1 in hepatocellular carcinoma

**DOI:** 10.18632/aging.202380

**Published:** 2021-01-20

**Authors:** Pengfei Zhu, Ruo Feng, Xu Lu, Yuan Liao, Zhicheng Du, Wenlong Zhai, Kunlun Chen

**Affiliations:** 1 Department of Hepatobiliary and Pancreatic Surgery, The First Affiliated Hospital of Zhengzhou University, Henan 450052, P.R. China; 2 Department of Histology and Embryology, School of Basic Medical Sciences, Zhengzhou University, Zhengzhou 450052, Henan, P.R. China

**Keywords:** AKR1C3, AKR1D1, diagnosis, prognosis, hepatocellular carcinoma

## Abstract

Hepatocellular carcinoma (HCC) is the most common histological type of primary liver cancer and the majority of patients are diagnosed at an advanced stage and have a poor prognosis. AKR1C3 (Aldo-keto reductase family 1 member C3) and AKR1D1 (Aldo-keto reductase family 1 member D1) catalyze the conversion of aldehydes and ketones to alcohols and play crucial roles in multiple cancers. However, the functions of AKR1C3 and AKR1D1 in HCC remain unclear. In our study, data from the public databases were selected as training and validation sets, then 76 HCC patients in our center were chosen as a test set. Bioinformatics methods suggested AKR1C3 was overexpressed in HCC and AKR1D1 was down-regulated. The receiver operating characteristic curve (ROC) analysis was performed and the area under curve (AUC) values of AKR1C3 and AKR1D1 were above 0.7 (0.948, 0.836, respectively). Also, the high expression of AKR1C3 and low expression of AKR1D1 predicted poor prognosis and short median survival time. Then, the knockdown of AKR1C3 and overexpression of AKR1D1 in HCC cells were achieved with lentivirus. And both decreased cell proliferation, restrained cell viability, and inhibited tumorigenesis. Moreover, the gene ontology (GO) and Kyoto Encyclopedia of Genes and Genomes (KEGG) enrichment analyses were conducted and the results showed that AKR1C3 and AKR1D1 might participate in the MAPK/ERK and androgen receptor (AR) signaling pathway. Furthermore, the AR and phosphorylated ERK1/2 were significantly reduced after the suppression of AKR1C3 or overexpression of AKR1D1. Collectively, AKR1C3 and AKR1D1 might serve as candidate diagnostic and prognostic biomarkers for HCC and provide potential targets for HCC treatment.

## INTRODUCTION

Liver cancer is the sixth most frequently diagnosed malignant tumor and the fourth leading cause of cancer-related deaths worldwide, with about 841,000 new cases and 782,000 deaths per annum [1]. Hepatocellular carcinoma (HCC) accounts for about 90% of cases of primary liver cancer and the major risk factor for HCC is hepatitis B virus infection [2, 3]. Currently, most HCC patients are diagnosed at advanced stages; thus, the 5-year relative survival rate of HCC is approximately 12% [4]. Therefore, novel biomarkers for HCC diagnostic accuracy and prognostic prediction are critically needed.

Aldo-keto reductase family 1 (AKR1) is a separate superfamily of proteins that participate in converting aldehydes and ketones to their corresponding alcohols by utilizing NADH or NADPH [5]. Previously, AKR1A1 was reported to be upregulated and associated with acquired radioresistance of laryngeal cancer [6]. AKR1B1 also acted as an oncogene, which formed a positive feedback loop and activated the EMT program in breast cancer [7]. Meanwhile, AKR1B10 induced the migration and invasion of breast cancer cells by promoting the expressions of MMP2 and Vimentin via activating EKR signaling [8]. Zhu and Zhang et al. (2018) found that AKR1C1 and AKR1C2 would promote the metastasis of non-small cell lung cancer via the JAK2/STAT3 axis [9]. Generated by hepatocytes and bile duct cells, AKR1C3 plays a crucial role in catalyzing the reduction of Δ4-androstene-3,17-dione to testosterone and promoting the generation of 17β-estradiol to regulate the activity of estrogen receptors [10]. Notably, the overexpression of AKR1C3 was observed in various metabolic diseases, hormone-related carcinomas, and drug resistance [11–13]. The hepatocytes express AKR1D1 and govern bile acids (BAs) production. Also, AKR1D1 generates all 5β-reduced metabolites for C19-, C21- and C27-steroids, including androgens and glucocorticoids. Previous studies showed that the dysregulation of AKR1D1 might lead to non-alcoholic fatty liver disease (NAFLD) and NAFLD was the most common reason for chronic liver disease in Western countries [14, 15]. Yet, few studies focus on the diagnostic and prognostic values of AKR1C3 and AKR1D1 in HCC. And the potential mechanisms remain unknown and patients with HCC might benefit from research on the two enzymes.

In the present study, the diagnostic and prognostic values of AKR1C3 and AKR1D1 were identified in HCC patients. Moreover, the hidden mechanism was further explored in HCC cell lines *in vitro*. Briefly, AKR1C3 and AKR1D1 might be promising biomarkers in the early diagnosis and prognosis evaluation of HCC patients. Furthermore, by integrating bioinformatics analysis and experimental validation, our study might provide novel and potential targets for the individual comprehensive therapy in HCC.

## RESULTS

### AKR1C3 was upregulated and AKR1D1 was downregulated in HCC

**Figure 1:**
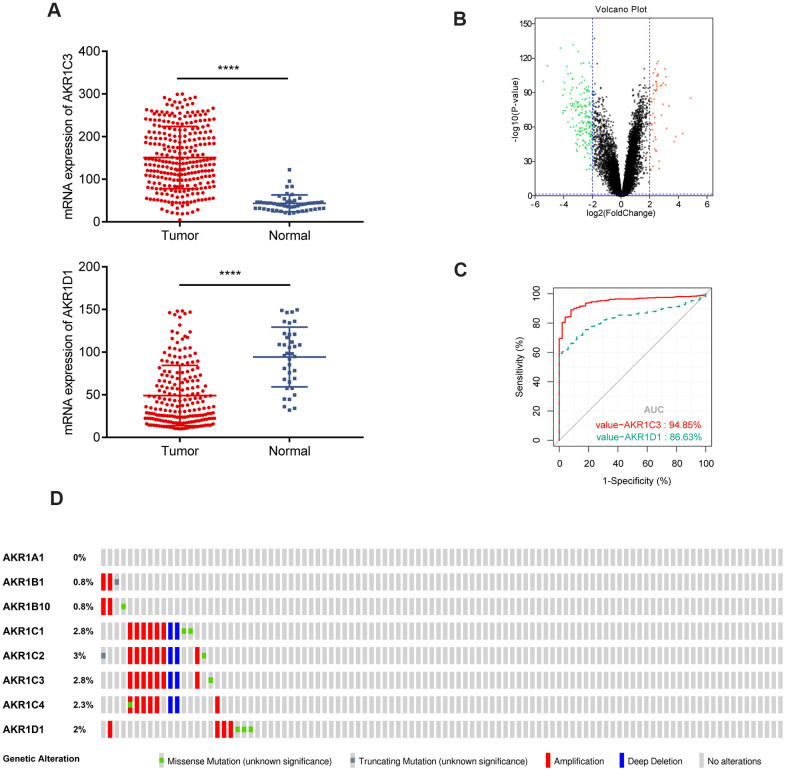
**Gene expression of AKR1C3 and AKR1D1 in training and validation sets.** (**A**) The mRNA expression of AKR1C3 and AKR1D1 in the training set. (**B**) The volcano plot of differential expressed genes in the validation set. (**C**) The receiver operating characteristic (ROC) curve of AKR1C3 and AKR1D1. (**D**) Genetic alteration information of the AKR1 gene family.

The scatter plots showed that AKR1C3 was upregulated and AKR1D1 was downregulated in the training set (Figure 1A). Meanwhile, AKR1C3 and AKR1D1 were also differentially expressed in the validation set (Figure 1B). To identify the diagnostic value of AKR1C3 and AKR1D1, the receiver operating characteristic (ROC) curve was visualized. And the results suggested that the area under curve (AUC) values of AKR1C3 and AKR1D1 were 0.948 and 0.836, which meant AKR1C3 and AKR1D1 had robust diagnostic abilities for HCC (Figure 1C). Furthermore, 2.8% of tumor samples showed AKR1C3 mutations, including amplifications, deep deletions, and missense mutations; 2% of the samples also showed genetic alterations in the AKR1D1 gene (Figure 1D).

### AKR1C3 is a fatal prognostic factor and AKR1D1 is a favorable one in HCC

**Figure 2:**
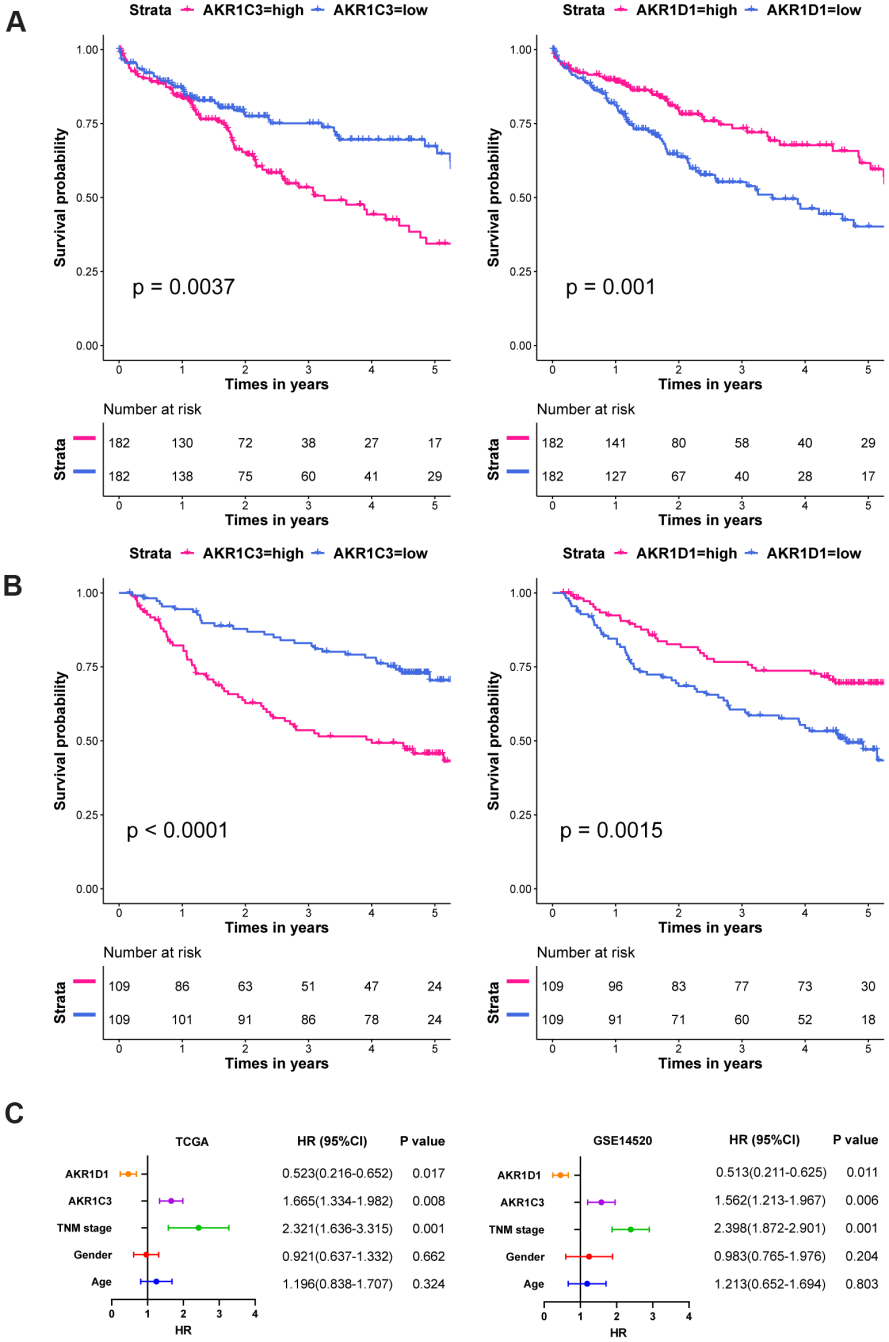
**The survival curves and multivariate COX regression of AKR1C3 and AKR1D1.** (**A**) The survival curves of AKR1C3 and AKR1D1 in the training set. (**B**) The survival curves of AKR1C3 and AKR1D1 in the validation set. (**C**) The multivariate COX regression of AKR1C3 and AKR1D1 in the training and validation sets.

In the survival analysis, high expression of AKR1C3 significantly associated with short median survival time and poor prognosis in both training and validation sets (Figure 2A, 2B, P value=0.0037 and P value<0.0001, respectively). Yet, the high expression of AKR1D1 suggested better overall survival than the low expression group in training and validation sets (P value=0.001 and P value=0.0015, respectively). In the subsequent multivariate analysis, the COX regression models were adjusted with age, gender, and TNM stage. And the results - (Figure 2C). Moreover, the detailed clinical characteristics of training and validation sets could be found in Supplementary Tables 1, 2.

### The prognostic values of AKR1C3 and AKR1D1 in subgroup and joint-effect analysis

**Figure 3:**
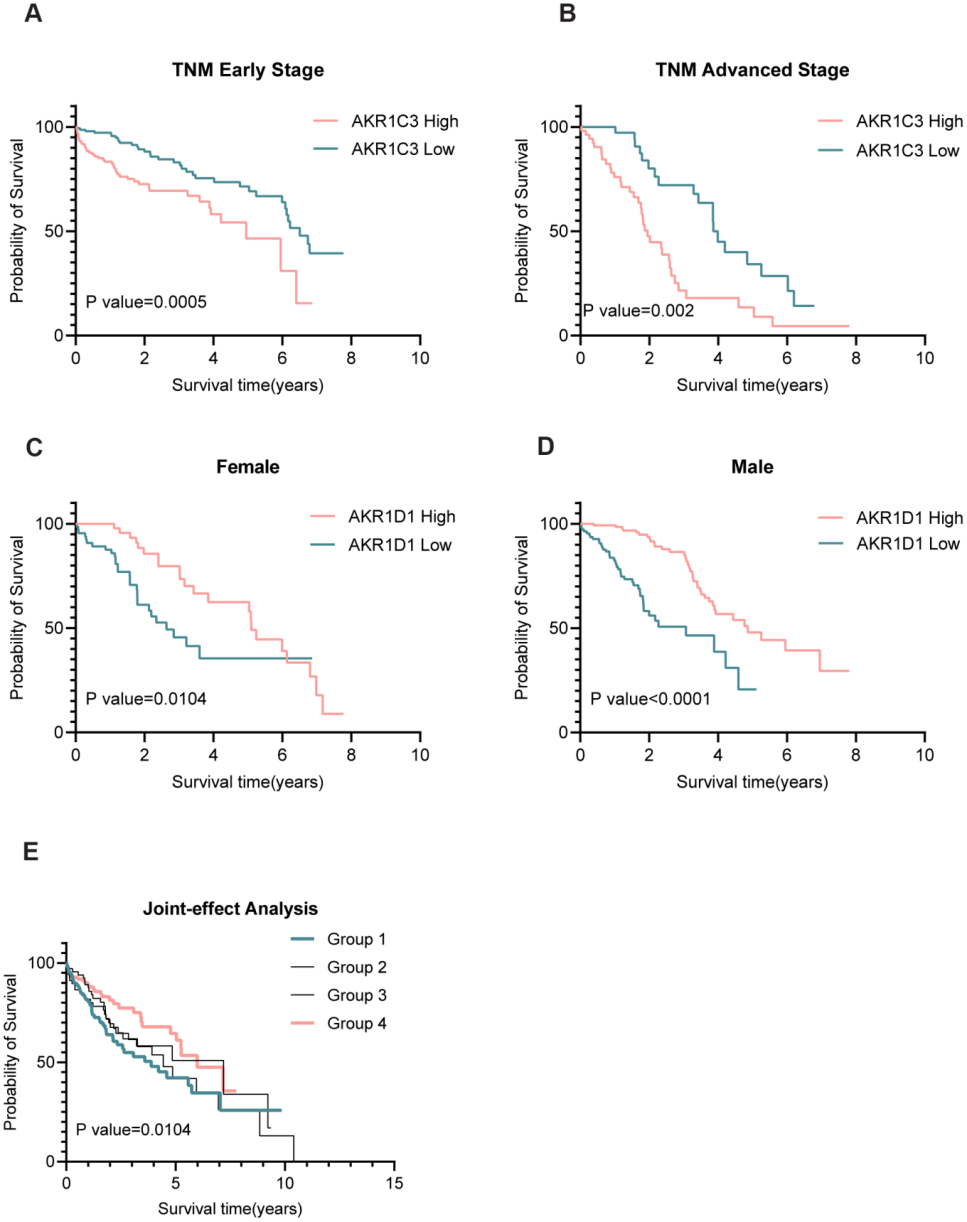
**Subgroup and joint-effect analysis of AKR1C3 and AKR1D1.** (**A**, **B**) High expression of AKR1C3 suggested poor prognosis in the early and advanced TNM stage. (**C**, **D**) Low expression of AKR1D1 was related to poor prognosis in male and female. (**E**) Joint-effect analysis of AKR1C3 and AKR1D1.

The results of Chi-square tests revealed that AKR1C3 was related to the TNM stage while AKR1D1 was associated with gender (Table 1). And the subgroup analysis suggested that high expression of AKR1C3 meant poor prognosis both in the early and advanced TNM stage (Figure 3A, 3B). Also, the low expression of AKR1D1 indicated short overall survival both in males and females (Figure 3C, 3D). Only AKR1C3 and AKR1D1 were significantly associated with prognosis in training and validation sets (Supplementary Figure 1), so we combined AKR1C3 and AKR1D1 to perform joint-effect analysis. And group 1 (high AKR1C3 and low AKR1D1) showed the worst prognosis, while group 4 (low AKR1C3 and high AKR1D1) showed the best prognosis (Figure 3E). All the results identified the prognostic values of AKR1C3 and AKR1D1.

**Table 1:** The chi-square test between characteristics and AKR1C3 (or AKR1D1).

**Variables**	**AKR1C3 Expression**		**P-value**	**AKR1D1 Expression**		**P-value**
	**High**	**Low**		**High**	**Low**	
Race			0.087			0.070
Asian	85	70		65	90	
White+others	93	105		109	89	
Gender			0.217			**0.017**
Male	55	63		49	69	
Female	127	119		133	113	
Age			0.458			0.086
<60	86	88		80	94	
≥60	96	94		102	88	
BMI			0.452			0.069
<25	88	87		79	96	
≥25	26	79		86	69	
TNM stage			**0.014**			0.243
Early	126	145		139	132	
Advanced	55	36		42	49	

BMI: Body Mass Index. TNM: Tumor, Node, Metastasis.

### Extra validation of AKR1C3 and AKR1D1 in the test set

**Figure 4:**
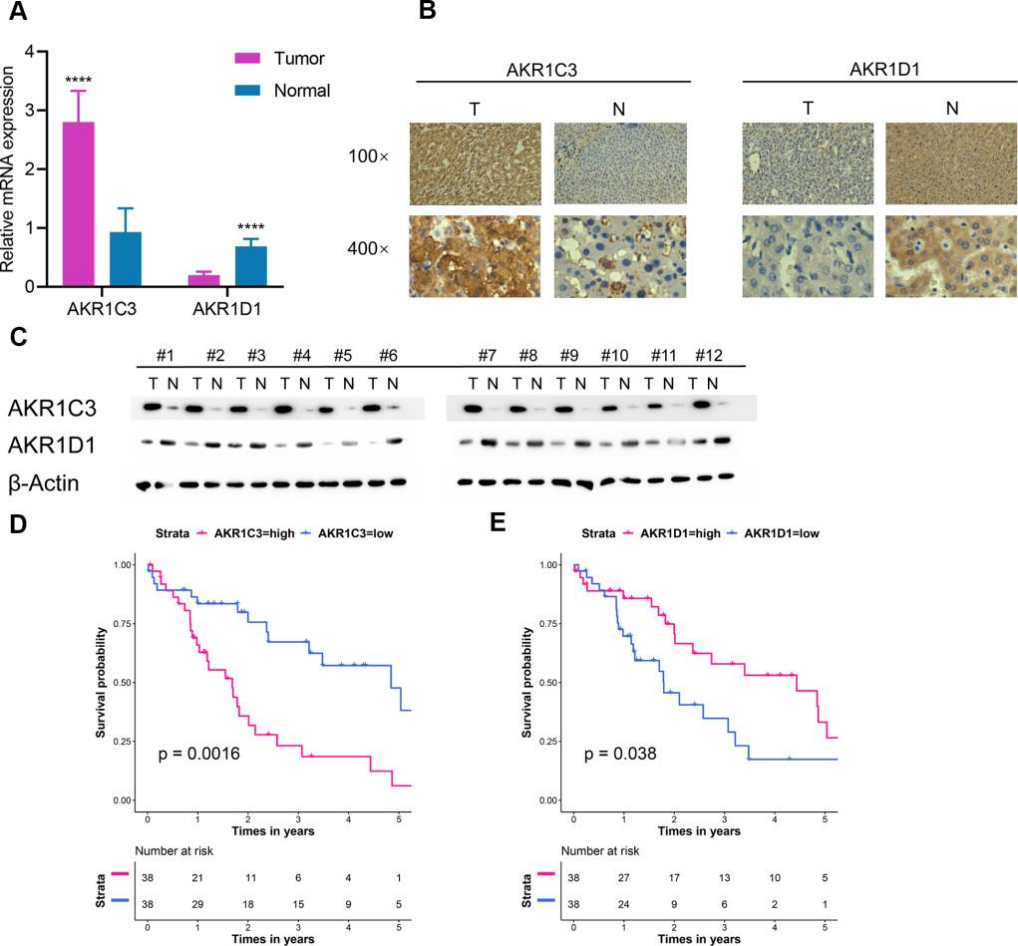
**Extra validation of AKR1C3 and AKR1D1 in the test set.** (**A**) Relative mRNA expression of AKR1C3 and AKR1D1 between tumor and normal. (**B**) The IHC staining results of AKR1C3 and AKR1D1. (**C**) The protein level of AKR1C3 and AKR1D1 between tumor and normal tissues. (**D**) The survival curve of AKR1C3 in the test set. (**E**) The survival curve of AKR1D1 in the test set.

76 paired HCC tumor and adjacent normal tissues in our center were collected and detailed clinical characteristics were recorded in Table 2. In normal tissue, AKR1D1 was secreted by hepatocyte and AKR1C3 was secreted by hepatocyte and bile duct. In tumor tissue, AKR1C3 and AKR1D1 were secreted by HCC cells. The mRNA and protein levels of AKR1C3 increased in HCC tumor tissues (Figure 4A–4C). And the expression of AKR1D1 was downregulated in tumor tissue. Further survival analysis identified the prognostic values of AKR1C3 and AKR1D1 (Figure 4D, 4E).

**Table 2:** Survival analysis of 76 HCC patients in the test set.

**Variables**		**Cases (n=76)**	**MST (years)**	**HR (95%CI)**	**Log-rank P**
Gender					**0.008**
Female		25	4.844	Ref	
Male		51	1.781	2.504(1.247-5.029)	
AFP					0.073
Low		16	1.687	Ref	
High		60	2.576	1.895(0.932-3.855)	
TNM stage					**0.007**
Early		33	6.369	Ref	
Advanced		43	3.237	1.547(1.118-2.142)	
Vascular invasion					**<0.001**
No		16	5.036	Ref	
Yes		60	1.823	5.812(2.156-7.644)	
AKR1C3					**0.001**
Low expression		38	4.653	Ref	
High expression		38	1.687	1.687(1.211-2.331)	
AKR1D1					**0.038**
Low expression		38	1.792	Ref	
High expression		38	4.436	0.576(0.305-0.889)	

HCC: hepatocellular carcinoma; MST: median survival time; AFP: alpha-fetoprotein; Ref: reference.

### AKR1C3 and AKR1D1 regulated the activity of MEK/ERK and AR signaling pathway

**Figure 5:**
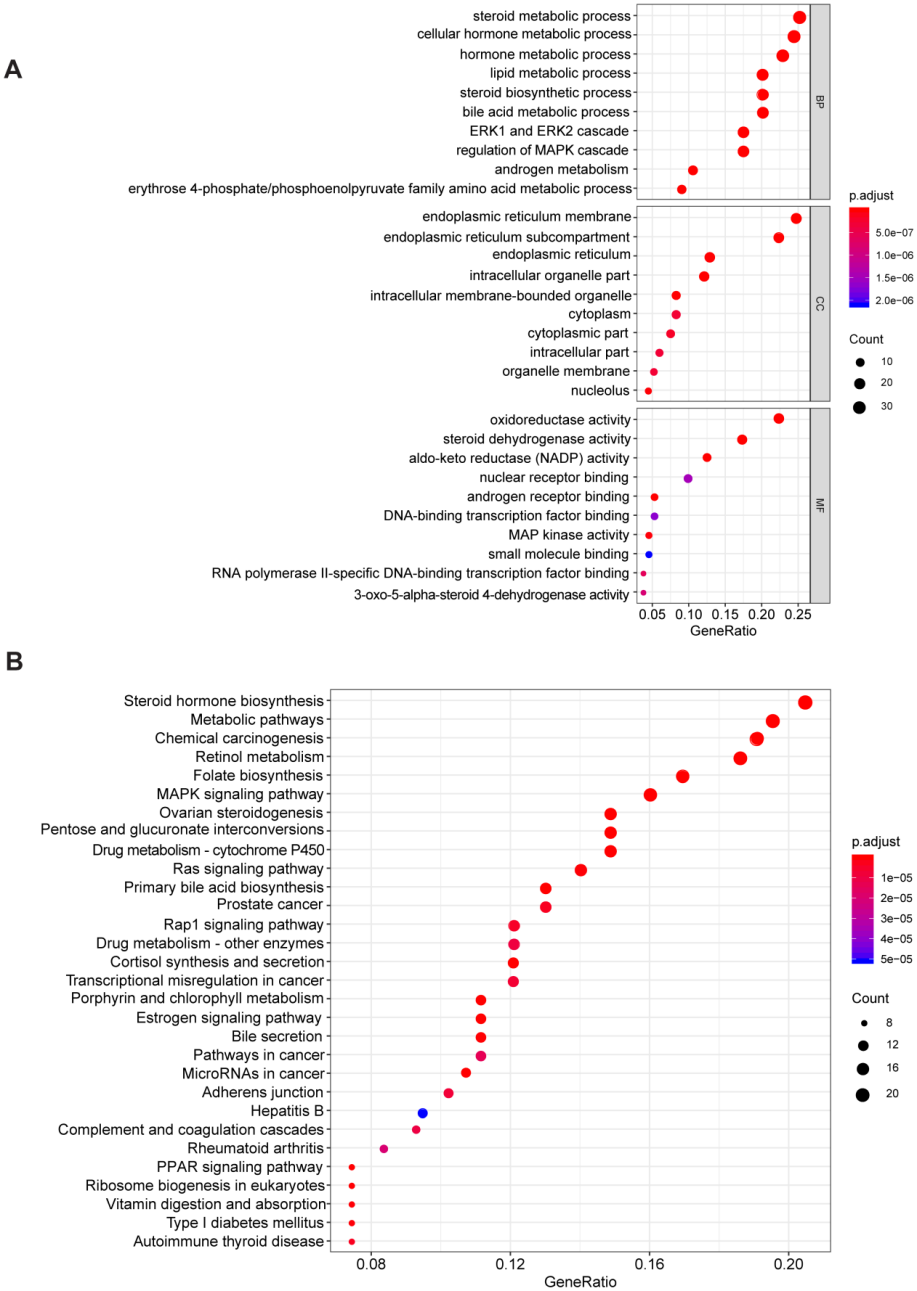
**The GO and KEGG enrichment analysis.** (**A**) The results of GO enrichment. (**B**) The results of KEGG enrichment.

**Figure 6:**
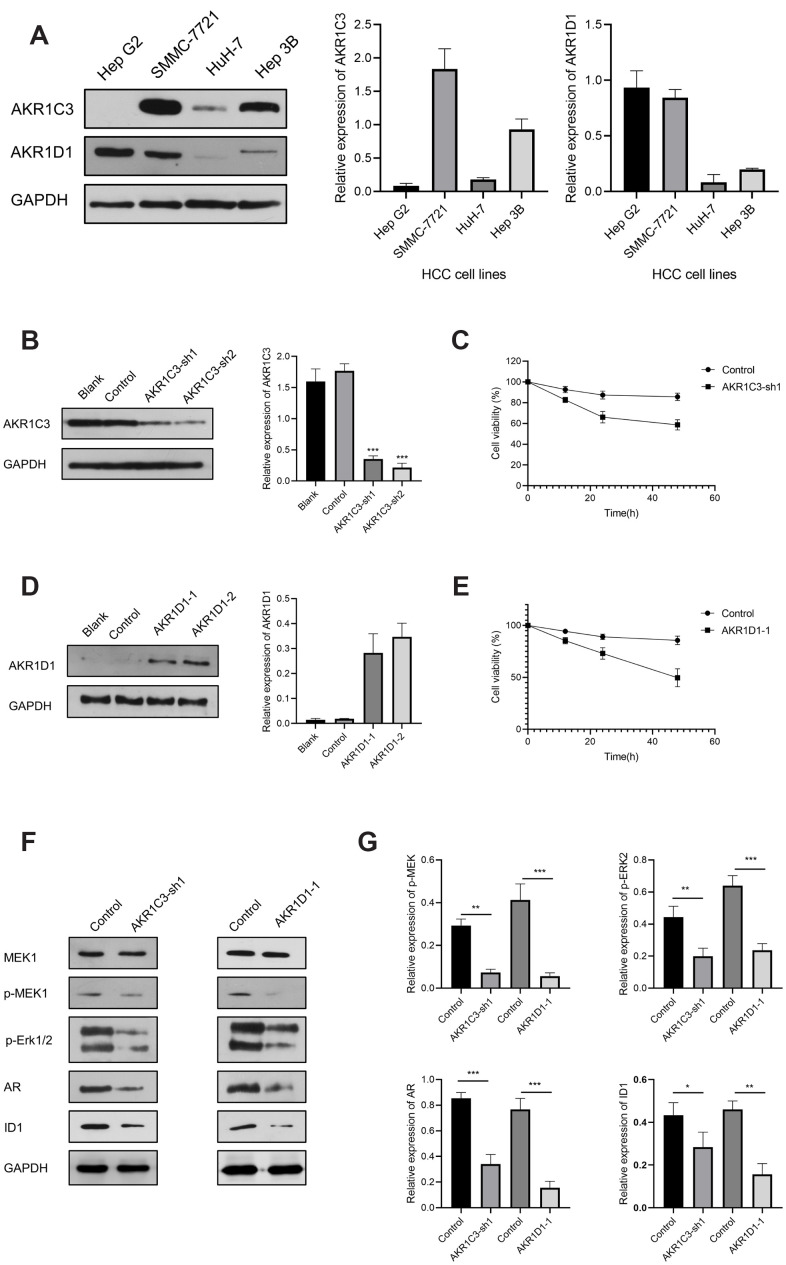
**The results of AKR1C3 and AKR1D1 lentiviral transfection.** (**A**) Relative protein expression of AKR1C3 and AKR1D1 in HCC cell lines. (**B**) The protein level of AKR1C3 in SMMC-7721 cells after the knockdown. (**C**) The cell viability of sh-AKR1C3 and control groups. (**D**) The protein level of AKR1D1 in HuH-7 cells after overexpression. (**E**) The cell viability of AKR1D1-1 and control groups. (**F**, **G**) The protein levels of MEK1, p-MEK1, p-Erk1/2, AR, and ID1 in the AKR1C3 knockdown and AKR1D1 overexpression cells.

To explore the potential mechanism of AKR1C3 and AKR1D1, protein-protein interaction (PPI) and enrichment analysis were initially conducted. The results of PPI revealed that AKR1C3 and AKR1D1 might interact with various key proteins, including MAPK1, MAPK3, MAP3K2, and AR (Supplementary Figure 2). Then, the GO and KEGG enrichment analysis suggested that AKR1C3 and AKR1D1 might regulate steroid metabolic process, bile acid metabolic process, MAPK cascade activity, and androgen metabolism (Figure 5A, 5B). Then, the protein levels of AKR1C3 and AKR1D1 were detected in HCC cell lines. And AKR1C3 had the highest expression in SMMC-7721, while AKR1D1 had the lowest expression in HuH-7 (Figure 6A). After the knockdown of AKR1C3 in SMMC-7721 cells, the cell viability significantly decreased and the overexpression of AKR1D1 in HuH-7 cells also inhibited cell proliferation (Figure 6B–6E). Further study revealed that the knockdown of AKR1C3 and overexpression of AKR1D1 decreased the p-MEK, p-Erk1/2, AR, and ID1 protein expression (Figure 6F, 6G). Our studies suggested that AKR1C3 and AKR1D1 might oppositely regulate MEK/Erk and AR signaling pathways.

## DISCUSSION

HCC still poses a serious public health burden worldwide, mainly owing to the lack of efficient indicators and effective treatment [16]. Most HCC patients are diagnosed at advanced stages and the tumors become unresectable [17]. Discoveries of novel biomarkers and molecular mechanisms are of great clinical significance for HCC treatment [18]. Here, our study identified the diagnostic and prognostic values of AKR1C3 and AKR1D1. Further mechanism research revealed that AKR1C3 and AKR1D1 might regulate MEK/ERK and AR signaling pathways oppositely.

Aldo-keto reductases (AKRs) are NADP(H) oxidoreductases and catalyze reactions on a wide-ranging spectrum of substrates, including drugs and carcinogens [19, 20]. AKR1C3 is a member of the AKR superfamily and acts as a drug target in hormonal malignancies and endocrine disorders [21]. Previous studies revealed that the expression of AKR1C3 increased in prostate cancer and estrogen receptor-positive breast cancer, which were associated with significantly decreased survival [22, 23]. And AKR1C3 promoted hormonal cancers progression by enhancing local androgen and estradiol production via the “backdoor” pathway and membrane-bound estrogen receptor GPER, respectively [24–26]. In the research by Wang Bin et al., AKR1C3 also acted as a novel driver of epithelial-to-mesenchymal transition in cancer metastasis through the activation of ERK signaling [27]. In the present study, AKR1C3 was found to be significantly upregulated in HCC (Figure 1A, 1B) and then identified in clinical samples (Figure 4B, 4C). High expression of AKR1C3 was an independent risk factor for HCC and related to poor prognosis (Figure 2). Our data suggested that AKR1C3 might act as an oncogene to accelerate the HCC progression. On the other hand, AKR1D1 was reported to catalyze fundamental steps in bile acid synthesis and inactivate steroid hormones [28, 29]. Nikolaou Nikolaos et al. demonstrated that AKR1D1 silencing promoted hepatocyte triglyceride accumulation by increasing lipogenesis and fueling hepatocyte inflammation in non-alcoholic fatty liver disease [14, 30]. Also, AKR1D1 over-expression decreased glucocorticoid production and glucocorticoid receptor activation in human hepatoma cells [15, 31]. In our study, the diagnostic effect of AKR1D1 was tested with ROC (AUC=0.866, Figure 1C). And the high expression of AKR1D1 was an independent factor in HCC and associated with longer median survival time (Figure 2). The results demonstrated that AKR1D1 might prevent liver carcinogenesis and this was in line with earlier research. Further bioinformatics analysis suggested that the two genes were significantly enriched in MEK/ERK and AR signaling pathway (Figure 5A), which provided some guidance for the following studies.

ERK1 and ERK2 were evolutionarily conserved, essential serine-threonine kinases, and known to activate multiple downstream targets, including EST1, FoxO3a, and c-Fos [32, 33]. And the aberrant activation of the MEK/ERK signaling pathway was reported to be involved in tumor proliferation, metastasis, drug resistance, and recurrence [33, 34]. For HCC patients, the phospho-ERK correlated to hepatitis C virus (HCV) infection, aggressive tumor behavior, and constituted an independent prognostic factor [35]. Early studies suggested that AR signaling pathway contributed to the hepatocarcinogenesis considering the male predominance in HCC [36–38]. Ao et al. reported that AR induced HCC cell migration and invasion by increasing ID1 expression and this process could be blocked by the AR antagonist Casodex [39]. Based on a study by Ma et al., the knockdown of AR promoted the DNA sensing and repairing system in HCC cells and enhanced p53-mediated cell apoptosis by increasing p53 expression at the transcriptional level [40]. In our study, the knockdown of AKR1C3 and overexpression of AKR1D1 both significantly inhibited HCC cell proliferation (Figure 6C, 6E). Moreover, the protein levels of phospho-MEK, phospho-ERK1/2, AR, and ID1 were significantly decreased after the knockdown of AKR1C3 or overexpression of AKR1D1 (Figure 6F). Thus, these results indicated that AKR1C3 served as an oncogene by activating MEK/ERK and AR signaling pathways, while AKR1D1 might suppress the two pathways.

Yet, there still several limitations to our study. First, the results should be validated in larger cohorts of patients. Also, more clinical information, including alcohol intake, smoking status, Child-Pugh score, vascular invasion, and intrahepatic metastasis, should be collected to make the findings more reliable and trustworthy.

Briefly, our data revealed that AKR1C3 and AKR1D1 played vital roles in the diagnosis and prognosis of HCC. Further mechanism research demonstrated that the knockdown of AKR1C3 or overexpression of AKR1D1 suppressed the MEK/ERK and AR signaling pathways in HCC cells. Therefore, our study suggested that AKR1C3 and AKR1D1 might be candidates for hepatocellular carcinoma targeted therapy.

## MATERIALS AND METHODS

### Ethics statement

The research protocol was approved by the Zhengzhou University First Affiliated Hospital Ethics Committee (Zhengzhou, China). Also, the experiments were conducted complying with the relevant regulations and the written informed consents were obtained from patients.

### Clinical specimens

A total of 76 HCC tumor and adjacent normal tissues were collected from The Department of Hepatobiliary and Pancreatic Surgery in The First Affiliated Hospital of Zhengzhou University from January 2013 to December 2019. And all patients did not receive immunotherapy or chemotherapy. Then the clinical specimens were immediately stored in the liquid nitrogen for further RT-PCR and western blotting assays.

### Immunohistochemical staining

Immunohistochemistry staining was performed as previously described [41]. The tumor and normal tissues were fixed with 10% formalin for 72 h, embedded in paraffin, and then and then antigen repaired. The following rabbit polyclonal antibodies were used: Anti-AKR1C3 (1:100, ab236656, Abcam, Cambridge, MA, USA) and Anti-AKR1D1 (1:100, ab254943, Abcam, Cambridge, MA, USA). The staining was visualized using 1% DAB under light microscopy.

### Data collection and process

The messenger RNA (mRNA) sequencing results of 364 liver hepatocellular carcinoma (LIHC) patients and 50 normal samples in The Cancer Genome Atlas (TCGA) were accessed from an online tool Fire Browse (http://www.firebrowse.org/). These data were normalized by Transcripts Per Kilobase of exon model per Million mapped reads (TPM) using the “edgeR” R package [42]. The GSE14520 dataset, which consisted of 218 HCC and 221 normal samples, were then obtained from the Gene Expression Omnibus (GEO) database. And the raw data were standardized and transformed with log2(x+1) to ensure harmonized criteria with the “affy” R package [43]. Moreover, the clinical and pathological information of 76 HCC patients in our center were also recorded.

Briefly, the TCGA dataset was selected as a training set, the GSE14520 dataset was selected as a validation set, and the 76 HCC patients in our center was chosen as a test set. We designed these three sets to verify the diagnostic and prognostic values of AKR1C3 and AKR1D1.

### Identification of differentially expressed genes (DEGs) and receiver operating characteristic (ROC) curve

In the training set (TCGA dataset), the mRNA expressions of AKR1C3 and AKR1D1 were tested between tumor and normal samples using an unpaired Student t-test. To assess the efficacy of AKR1C3 and AKR1D1 in diagnosing HCC, we performed ROC analysis to investigate how large the area under the curve (AUC) was. Meanwhile, the genetic alteration rates of AKR1C3 and AKR1D1 were identified by accessing the cBioPortal for cancer genomics [44]. In the validation set, DEGs were obtained with the “Limma” R package in Rstudio software (version 1.3.959). And the cutoff values were set as follows: the adjusted P-value < 0.05 and |log2 fold change (FC)| > 2.

### Survival analysis and multivariate COX regression

Initially, the training set (TCGA dataset) was equally divided into the high-expression group (n=182) and low-expression group (n=182) based on the median of AKR1C3 and AKR1D1. And the Kaplan-Meier method was used to estimate the differences between the high and low-expression groups with “survival” and “survminer” R package. Then, the multivariate analyses were analyzed by the Cox regression model, which was adjusted with gender, age, and TNM stage. Also, the validation set (GSE14520) was split into the high-expression group (n=109) and low-expression group (n=109), and then the survival analyses were repeated for accuracy.

### Subgroup analysis and joint-effect analysis

To explore the relationship between gene expression and tumor biological behavior, chi-square tests were performed between AKR1C3 (AKR1D1) and other characteristics. Further, the TNM stage and gender were stratified and survival analyses were conducted. Subsequently, group 1 (high AKR1C3, low AKR1D1), group 2 (high AKR1C3, high AKR1D1), group 3 (low AKR1C3, low AKR1D1), and group 4 (low AKR1C3, high AKR1D1). Then, the overall survival in the four groups was assessed using the Kaplan-Meier method.

### Protein-protein interaction, GO and KEGG analysis

To uncover underlying mechanisms, we implemented protein-protein interaction (PPI), Gene Ontology (GO), and the Kyoto Encyclopedia of Genes and Genomes (KEGG) enrichment analysis with STRING database and “clusterprofiler” R package. In the PPI analysis, the potential interactions with AKR1C3 and AKR1D1 were predicted under the conditions as follows: high confidence (interaction score>0.400) and no more than 20 interactors per shell. Furthermore, the results of PPI analysis were applied in GO and KEGG enrichment analyses and the outcome was visualized by Cytoscape (Version 3.72) and Rstudio (Version 1.2.5033).

### Cell lines and culture

The hepatocellular carcinoma cell lines Hep G2, Hep 3B, and Huh-7 were obtained from the Cell Bank of the Chinese Academy of Sciences (Shanghai, China) and the SMMC-7721 cell line was a kind gift from professor Ye (Zhengzhou University, China). The cells were incubated in RPMI-1640 medium (Beyotime Institute of Biotechnology, Shanghai, China) or Dulbecco's Modified Eagle Medium (DMEM, Solarbio Life Science, Beijing, China) supplemented with 10% fetal bovine serum (FBS; HyClone, Utah, USA), 100 U/mL penicillin, 100 mg/L streptomycin in an incubator (Thermo Fisher Scientific, USA) at 37° C and 5% CO_2_. The cells used for experiments were in the exponential growth phase.

### Cell transfection

The lentiviruses were designed and purchased from GeneChem (Shanghai, China), including blank lentivirus, control lentivirus, lentivirus to knockdown AKR1C3 (shAKR1C3), and lentivirus to overexpress AKR1D1 (lv-AKR1D1). Then the SMMC-7721 and HuH-7 cells were seeded into 6-well plates and infected with the lentivirus according to the manufacturer’s instruction (MOI=10). Finally, the blank, control, sh-AKR1C3, and lv-AKR1D1 cells were obtained for further study.

### Cell viability assay

The cell viability assay was detected by CCK-8 assay. Briefly, the cells (5×10^3^/well) were seeded into the 96-well plates and 10 μL CCK-8 were added to each well at 0, 12, 24, and 48h. Then, the cells were cultured for another 2h and the optical density (OD) at 450 nm was measured by Varioskan LUX Multimode Microplate Reader (Thermo Fisher Scientific, USA). The cell viability was calculated from three independent experiments.

### RT-PCR assay

Total cellular RNA from tumor and normal tissues were isolated with Trizol reagent (Invitrogen) as previously described [45]. After converted to cDNA using the HI Script® Q RT Super Mix for qPCR (Vazyme), the RT-PCR was performed with SYBER green PCR master mix (Thermo Fisher Scientific). The primers used are listed below: GTCATCCGTATTTCAACCGGAG(AKR1C3, Forward, 5' -> 3'), CCACCCATCGTTTGTCTCGTT(AKR1C3, Reverse, 5' -> 3'), TCAGAACCTAAATCGACCCCT(AKR1D1, Forward, 5' -> 3'), TCCCCAACTTCGTGTTCATTTT(AKR1D1, Reverse, 5' -> 3'), GGAGCGAGATCCCTCCAAAAT(GAPDH, Forward, 5' -> 3'), and GGCTGTTGTCATACTTCTCATGG(GAPDH, Reverse, 5' -> 3').

### Western blot analysis

Tumor cells were lysed with RIPA Lysis Buffer and Phenylmethanesulfonylfluoride (PMSF, Beyotime Institute of Biotechnology, China), followed by denaturation at 100° C. The BCA Protein Assay Kit (Beyotime Institute of Biotechnology, China) was applied to determine the protein concentrations of samples. An equal amount of protein was loaded, separated by 10% SDS-PAGE gel, and blotted onto a nitrocellulose membrane. Then, the membrane was trimmed and blocked by 5% skim milk at room temperature for 2 h and incubated with primary antibodies at 4° C overnight. The antibodies include anti-AKR1C3 (ab236656, Abcam, Cambridge, MA, USA), anti-AKR1D1 (ab254943, Abcam, Cambridge, MA, USA), anti-MEK1 (12671, Cell Signaling Technology Inc, CST, MA, USA), anti-p-MEK1 (9127, Cell Signaling Technology Inc, CST, MA, USA), anti-p-Erk1/2 (4370, Cell Signaling Technology Inc, CST, MA, USA), anti-AR (19672, Cell Signaling Technology Inc, CST, MA, USA), anti-ID1 (ab168256, Abcam, Cambridge, MA, USA), and anti-GAPDH (5174, Cell Signaling Technology Inc, CST, MA, USA). Then the membranes were washed with TBST solution four times and incubated with the HRP-conjugated secondary antibody for 1 h at temperature. The protein band was finally incubated with enhanced chemiluminescence and exposed to an X-ray film.

### Statistical analysis

Results of the data were expressed as mean ± SD and statistical differences in two groups were performed by Student’s t-test. Data in the experiments were collected from three repeated experiments. And all data were processed using SPPS software (version 23.0, Chicago, IL, USA) and visualized with GraphPad Prism (version 8.0). P-value < 0.05 or less was considered statistically significant unless otherwise specified.

### Availability of data and materials

The data generated and analyzed during the current study are available from the corresponding author on a reasonable request.

## Supplementary Material

Supplementary Figures

Supplementary Tables
