# Supplementary material for: Diagnostic and prognostic values of AKR1C3 and AKR1D1 in hepatocellular carcinoma
Source: Aging (Albany NY). 2021 Jan 20;13(3):4138–56. doi: 10.18632/aging.202380 (PMC7906155; doi:10.18632/aging.202380)
Supplement: Supplementary Figures [file aging-13-202380-s001.pdf]

SUPPLEMENTARY FIGURES

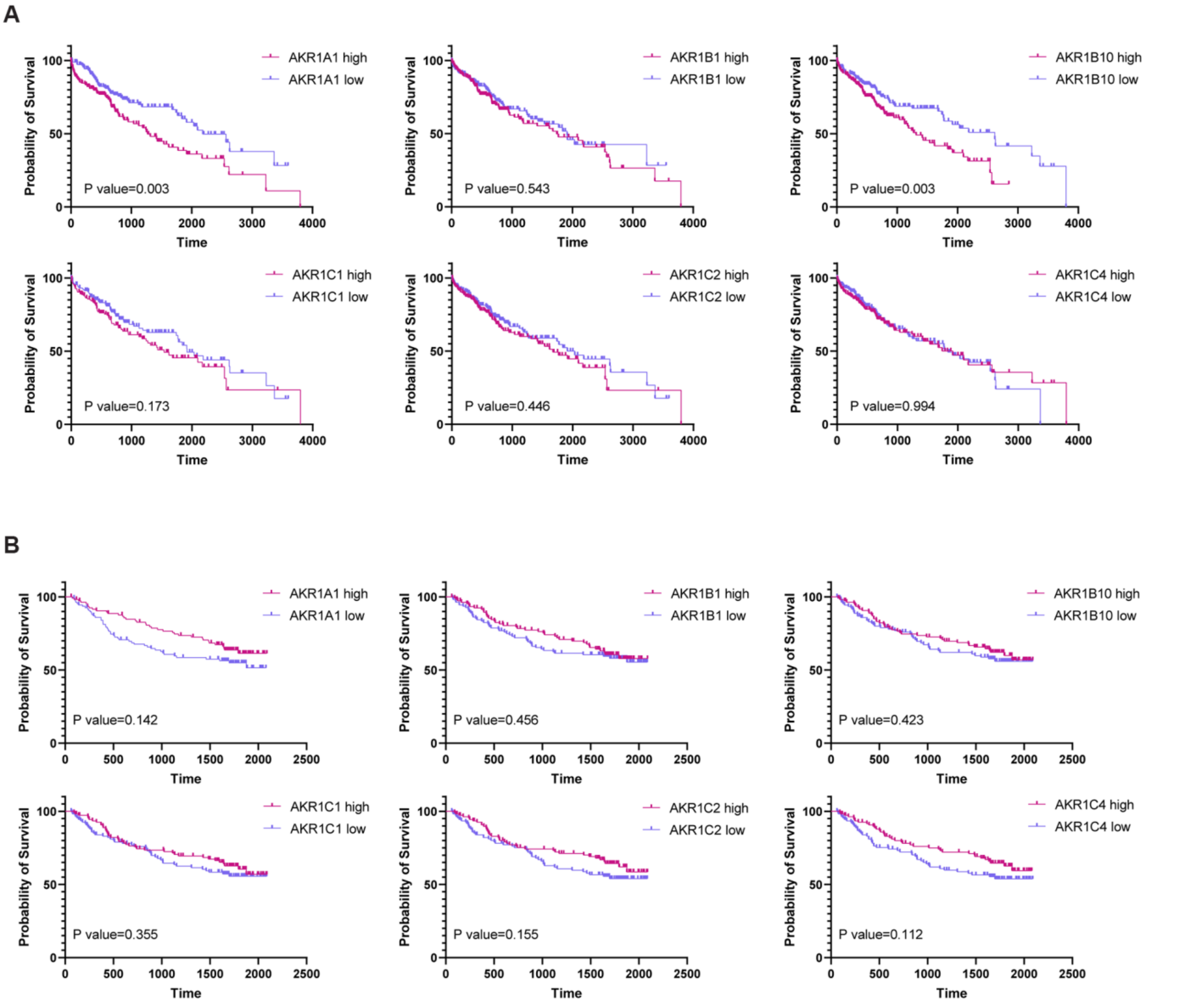

Supplementary Figure 1. The survival analysis of other AKR1s in the training (**A**) and validation (**B**) sets.

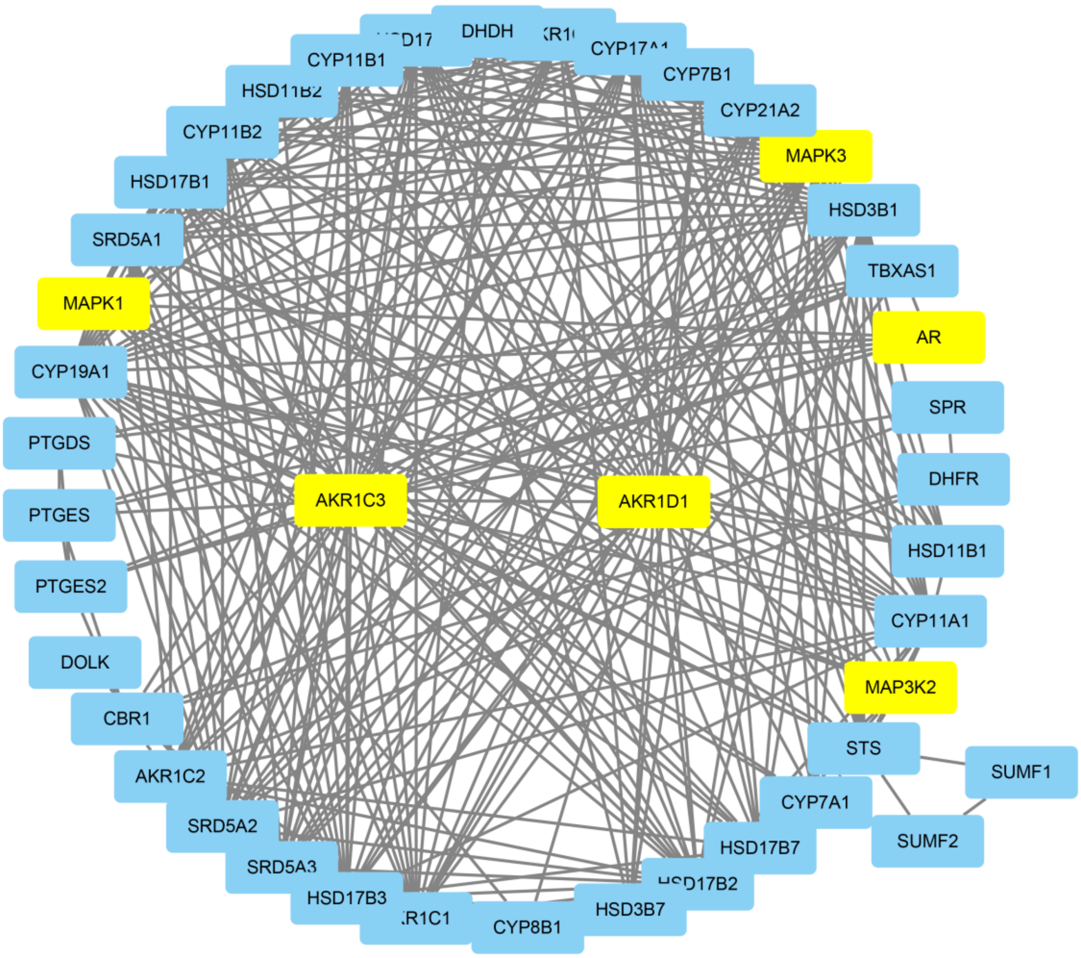

Supplementary Figure 2. **The results of protein-protein interaction (PPI) analysis.**
