# Supplementary material for: Diagnostic and prognostic values of AKR1C3 and AKR1D1 in hepatocellular carcinoma
Source: Aging (Albany NY). 2021 Jan 20;13(3):4138–56. doi: 10.18632/aging.202380 (PMC7906155; doi:10.18632/aging.202380)
Supplement: Supplementary Tables [file aging-13-202380-s002.pdf]

SUPPLEMENTARY TABLES

Supplementary Table 1. Clinical characteristics of HCC patients in the training set (TCGA).

Variables	Patients (n=364)	No. of events (%)	MST (months)	Log-rank P
Race				0.127
Asian	157	44(28.0%)	82	
White and others	207	81(40.1%)	68	
Gender				0.488
Male	243	79(31.7%)	57	
Female	121	51(42.1%)	59	
Age				0.248
≤60	171	55(31.1%)	71	
>60	193	75(38.9%)	52	
BMI				**0.021**
<25	177	61(34.5%)	69	
≥25	187	51(32.5%)	66	
TNM stage				**<0.001**
I+II	274	76(27.7%)	84	
III+IV	90	54(57.4%)	26	
Family history				0.192
Yes	112	49(44.1%)	40	
No	252	69(33.3%)	62	

MST, median survival time; BMI: Body Mass Index. TNM: Tumor, Node, Metastasis.

Supplementary Table 2. Clinical characteristics of HCC patients in the validation set (GSE14520).

Variables	Patients (n=218)	No. of events (%)	MST (months)	Log-rank P
Gender				0.149
Female	30	14(6.3%)	55	
Male	188	53(23.9%)	48	
Age				0.903
<60	178	79(35.7%)	49	
≥60	40	21(9.5%)	48	
HBV status				0.449
AVR-CC	56	35(15.8%)	45	
CC+NO	162	75(33.9%)	50	
ALT				0.726
<50u/L	127	61(27.6%)	49	
≥50u/L	91	51(23.0%)	49	
Main tumor size				**0.003**
<5cm	140	36(16.3%)	53	
≥5cm	81	54(24.4%)	41	
Multinodular				0.055
Yes	45	19(8.7%)	37	
No	173	69(31.6%)	47	
Cirrhosis				**0.019**
Yes	200	73(33.4%)	48	
No	18	9(4.1%)	64	
TNM stage				**<0.001**
I+II	169	33(15.1%)	54	
III	49	38(17.4%)	30	

BCLC stage				**<0.001**
0+A	167	36(16.5%)	55	
B+C	51	41(18.8%)	30	
AFP				**0.032**
<300ng/ml	118	36(16.5%)	53	
≥300ng/ml	100	53(24.3%)	44	

MST, median survival time; HBV, hepatitis B virus; ALT, alanine aminotransferase; AFP, alpha fetoprotein; BCLC stage, Barcelona Clinic Liver Cancer; TNM, Tumor, Node, Metastasis.
